# Endemic Melioidosis in Residents of Desert Region after Atypically Intense Rainfall in Central Australia, 2011

**DOI:** 10.3201/eid2106.141908

**Published:** 2015-06

**Authors:** Teem-Wing Yip, Saliya Hewagama, Mark Mayo, Erin P. Price, Derek S. Sarovich, Ivan Bastian, Robert W. Baird, Brian G. Spratt, Bart J. Currie

**Affiliations:** Northern Territory Centre For Disease Control, Alice Springs, Northern Territory, Australia (T-W. Yip);; Alice Springs Hospital, Alice Springs (S. Hewagama);; Menzies School of Health Research, Casuarina, Northern Territory, Australia (M. Mayo, E.P. Price, D.S. Sarovich, B.J. Currie);; Royal Adelaide Hospital, Adelaide, South Australia, Australia (I. Bastian);; South Australia Pathology, Adelaide (I. Bastian);; Royal Darwin Hospital, Casuarina (R.W. Baird, B.J. Currie);; Imperial College, London, UK (B.G. Spratt)

**Keywords:** Melioidosis, Burkholderia pseudomallei, endemic diseases, bacteria, rainfall, floods, desert, climate, Australia

## Abstract

After heavy rains and flooding during early 2011 in the normally arid interior of Australia, melioidosis was diagnosed in 6 persons over a 4-month period. Although the precise global distribution of the causal bacterium *Burkholderia pseudomallei* remains to be determined, this organism can clearly survive in harsh and even desert environments outside the wet tropics.

Melioidosis, a tropical disease caused by the bacterium *Burkholderia pseudomallei*, is endemic to Southeast Asia and northern Australia and is being increasingly recognized in other locations globally (*1*,*2*). During 1989–2009, a total of 540 cases of melioidosis were documented in a prospective melioidosis study based at Royal Darwin Hospital (latitude 12.4°S) in the tropical north of the Northern Territory of Australia (*3*). During that 20-year period, 4 of the study participants were considered to have been infected in the central Australia region of the Northern Territory; the other 536 were infected in the tropical north. During the same period, 2 persons who were not included in that study tested positive for *B. pseudomallei* in the central Australian region and were treated at Alice Springs Hospital (23.8°S), making a total of 6 cases of melioidosis during 20 years attributed to *B. pseudomallei* infection acquired in Central Australia. The ongoing Darwin prospective melioidosis study is approved by the Human Research Ethics Committee of the Northern Territory Department of Health and Menzies School of Health Research (approval 02/38).

## The Study

Central Australia (also known as the “Red Centre”) is the most inland part of the arid interior of Australia and features low average annual rainfall, a desert environment, and rivers that are often dry. During March–July 2011, a total 6 cases of melioidosis were diagnosed in patients from Central Australia who resided south of latitude 20°S (Figure) and had not traveled to the tropical north of the country or to overseas regions to which melioidosis is endemic. These cases occurred after exceptionally heavy rainfall in February and March 2011, which resulted in widespread flooding in this normally parched region. The heavy rainfall in Central Australia was linked to a La Niña–associated, record-breaking wet season during 2010–2011 in the tropical north of the Northern Territory (Figure). Tropical Cyclone Carlos formed over the Beagle Gulf north of Darwin on February 15. At Darwin airport, the total rainfall during February 2011 of 1,110.2 mm and during the October–April wet season of 2,926.8 mm were the highest on record for that weather station. At Alice Springs airport, rainfall was 107.6 mm during February 2011, compared with a February mean of 40.3 mm during 1981–2010. The February 2011 rainfall at Tennant Creek airport (19.6°S) was 309.6 mm, compared with a February mean of 122.3 mm during 1981–2010. During the month of March 2011, when the first 3 patients became ill, rainfall amounts at Alice Springs and Tennant Creek airports were 120.6 mm and 222.6 mm, respectively; the annual mean March rainfalls in these locations during 1981–2010 were 35.4 mm and 53.8 mm, respectively.

**Figure:**
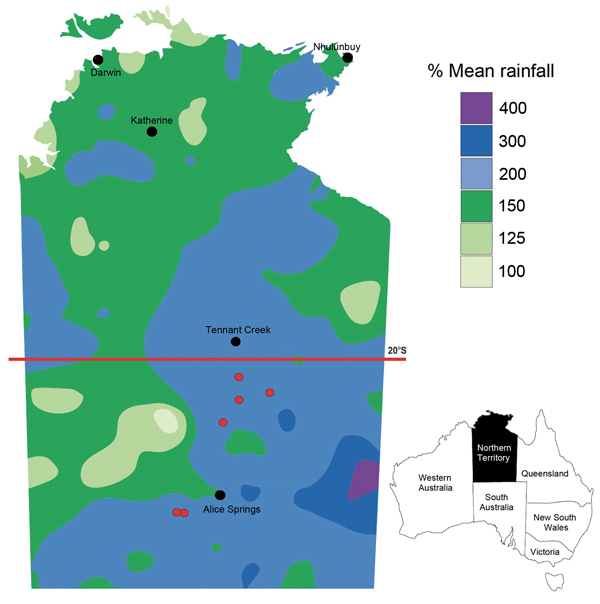
Rainfall in the Northern Territory of Australia during August 1, 2010–July 31, 2011. Rainfall is expressed as percentages of historical mean; the lowest rainfall total was 100% of mean. Cities and towns are indicated by black dots; locations of 6 persons with melioidosis in central Australia are indicated by red dots. Adapted from the National Climate Centre, Australian Bureau of Meteorology (http://www.bom.gov.au). Inset shows location of Northern Territory in Australia.

The 6 persons whose illnesses were diagnosed as endemic melioidosis were treated at the central Australia hospitals in Alice Springs and Tennant Creek (Table). Of the 6 patients, 3 were male and 5 were indigenous Aboriginal Australians; 3 became bacteremic and required intubation and ventilation in intensive care for severe sepsis. The only patient <50 years of age and no identified risk factor had the mildest disease, characterized by a localized skin abscess. All patients were treated according to standard guidelines (*1*): with initial intravenous ceftazidime or meropenem, then with oral eradication therapy, which is usually with trimethoprim/sulfamethoxazole. All 6 patients survived. 

**Table:** Details of 6 residents of desert region in whom melioidosis was diagnosed after heavy rainfall in Central Australia, 2011*

Age, y, sex, ethnicity	Month of illness onset	Risk factors	Clinical manifestation	*B. pseudomallei* culture source	MLST sequence type
22y, F, indigenous	March	Hazardous alcohol use	Brain abscess	Abscess pus	ST 897
32y, M, indigenous	March	Chronic renal disease	Axillary abscess	Abscess pus	ST 894
68y, F, indigenous	March	Elderly	Pneumonia, septic shock	Blood, sputum	ST 903
36 y, M, indigenous	April	Type 2 diabetes mellitus	Septic arthritis, septic shock	Blood, joint aspirate	ST 904
44 y, F, indigenous	May	Type 2 diabetes mellitus	Septic shock, no focus	Blood	ST 905
23y, M, caucasian	July	None	Skin abscess	Skin swab sample	ST 907
*Indigenous, Aboriginal people of Australia; MLST, multilocus sequence typing.					

Rainfall in the region returned to normal patterns and there have been no further locally acquired cases of melioidosis in Central Australia. Two persons with confirmed melioidosis were treated at Alice Springs Hospital in August 2011 and December 2014; both were attributed to infection acquired while traveling to the tropical north of the Northern Territory.

*B. pseudomallei* isolates from the 6 patients were subjected to multilocus sequence typing (MLST) as described by Godoy et al (*4*). On interrogation of the global *B. pseudomallei* MLST database (http://bpseudomallei.mlst.net/), each of the 6 isolates was unique and a novel sequence type (ST) (Table). ST907 has 2 novel alleles (gmhD, 54 and lipA, 48) and ST905 has 1 novel allele (lepA, 44); the remaining 4 STs contained unique configurations of existing alleles. None of the 6 STs was a single-locus variant of any ST in the MLST database (i.e., sharing 6/7 alleles), but all in the database except ST904 had double locus variants (i.e., sharing 5/7 alleles) that were *B. pseudomallei* isolates from northern Australia. Subsequent environmental soil sampling from Central Australia has confirmed the endemic presence of *B. pseudomallei* in several locations, and further studies are planned to characterize the environmental correlates of such *B. pseudomallei*–positive sites in Central Australia (M. Mayo, B.J. Currie, unpub. data).

Historically, melioidosis has been found to occur in the wet tropics between latitudes 20°S and 20°N (*5*,*6*). Nevertheless, the first recognition of melioidosis in Australia was in an outbreak among sheep in 1949 at Winton, Queensland (22.4°S) (*7*), an arid location with geographic similarities to Central Australia and where flooding also sporadically occurs. Case clusters of melioidosis have also occurred even further south in Australia: 1 cluster spanned 25 years in temperate southwestern Western Australia (31°S) (*8*). Severe weather events with heavy rainfall have dramatically increased the case numbers in regions in Australia and overseas to which the organism is endemic (*9*,*10*) and have also unmasked melioidosis in locations where it was uncommon or not previously recognized as being endemic (*11*–*13*).

Although genotype profiles showed the 6 isolates to be closer to STs of other Australian *B. pseudomallei* than to STs from Southeast Asia and the rest of the world, the extensive diversity among *B. pseudomallei* in Central Australia is evident in that each isolate is a novel ST and that none has any known single-locus variants. This diversity is similar to the situation seen in tropical northern Australia and in northeast Thailand (*14*) and contrasts with endemic melioidosis identified in Puerto Rico, which was recently shown to be mostly restricted to a single ST (*15*). The limited genetic diversity of *B. pseudomallei* seen in Puerto Rico to date is consistent with more recent introduction of *B. pseudomallei* to that location, potentially through importation of infected animals, as has occurred elsewhere (*2*). Importation of infected animals from the tropical north of Australia was also considered a possible source of the clonal cluster of melioidosis seen in various animals and in a person in southwestern Western Australia (*8*).

Although the presence of *B. pseudomallei* in central Australia is clearly not a recent phenomenon, the origins and longevity of *B. pseudomallei* in the region and the phylogenetic relationships to *B. pseudomallei* in tropical Australia and elsewhere globally require further studies using whole genome sequencing. Furthermore, the geographic boundaries of *B. pseudomallei* across the vast interior of the Australian continent and the extent of incursion into southern Australia remain entirely unclear.

## Conclusions

Heavy rains and flooding in the normally arid interior of Australia early in 2011 were followed by 6 cases of melioidosis over a 4-month period. Although the true extent of the environmental presence of *B. pseudomallei* remains to be determined, both regionally and globally (*1*,*2*), these bacteria can clearly survive in harsh and even desert environments outside the traditionally recognized melioidosis-endemic regions of the wet tropics. Melioidosis should therefore be considered in such regions, especially after heavy rainfall and flooding.

## References

[1] Wiersinga WJ, Currie BJ, Peacock SJ. Melioidosis. N Engl J Med. 2012;367:1035–44 . 10.1056/NEJMra1204699 22970946

[2] Dance DA. Melioidosis in Puerto Rico: the iceberg slowly emerges. Clin Infect Dis. 2015;60:251–3. 10.1093/cid/ciu768 25270648 PMC4275060

[3] Currie BJ, Ward L, Cheng AC. The epidemiology and clinical spectrum of melioidosis: 540 cases from the 20 year Darwin prospective study. PLoS Negl Trop Dis. 2010;4:e900 . 10.1371/journal.pntd.0000900 21152057 PMC2994918

[4] Godoy D, Randle G, Simpson AJ, Aanensen DM, Pitt TL, Kinoshita R, Multilocus sequence typing and evolutionary relationships among the causative agents of melioidosis and glanders, *Burkholderia pseudomallei* and *Burkholderia mallei.* J Clin Microbiol. 2003;41:2068–79. 10.1128/JCM.41.5.2068-2079.2003 12734250 PMC154742

[5] Cheng AC, Currie BJ. Melioidosis: epidemiology, pathophysiology, and management. Clin Microbiol Rev. 2005;18:383–416. 10.1128/CMR.18.2.383-416.2005 15831829 PMC1082802

[6] Dance DA. Melioidosis: the tip of the iceberg? Clin Microbiol Rev. 1991;4:52–60 . 2004347 10.1128/cmr.4.1.52 PMC358178

[7] Cottew GS. Melioidosis in sheep in Queensland; a description of the causal organism. Aust J Exp Biol Med Sci. 1950;28:677–83. 10.1038/icb.1950.70 14838792

[8] Currie B, Smith Vaughan H, Golledge C, Buller N, Sriprakash KS, Kemp DJ. *Pseudomonas pseudomallei* isolates collected over 25 years from a non-tropical endemic focus show clonality on the basis of ribotyping. Epidemiol Infect. 1994;113:307–12. 10.1017/S0950268800051736 7523158 PMC2271530

[9] Lo TJ, Ang LW, James L, Goh KT. Melioidosis in a tropical city state, Singapore. Emerg Infect Dis. 2009;15:1645–7. 10.3201/eid1510.090246 19861063 PMC2866399

[10] Parameswaran U, Baird RW, Ward LM, Currie BJ. Melioidosis at Royal Darwin Hospital in the big 2009–2010 wet season: comparison with the preceding 20 years. Med J Aust. 2012;196:345–8. 10.5694/mja11.11170 22432675

[11] Ko WC, Cheung BM, Tang HJ, Shih HI, Lau YJ, Wang LR, Melioidosis outbreak after typhoon, southern Taiwan. Emerg Infect Dis. 2007;13:896–8. 10.3201/eid1306.060646 17553230 PMC2792857

[12] Munckhof WJ, Mayo MJ, Scott I, Currie BJ. Fatal human melioidosis acquired in a subtropical Australian city. Am J Trop Med Hyg. 2001;65:325–8 . 11693877 10.4269/ajtmh.2001.65.325

[13] Christenson B, Fuxench Z, Morales JA, Suarez-Villamil RA, Souchet LM. Severe community-acquired pneumonia and sepsis caused by *Burkholderia pseudomallei* associated with flooding in Puerto Rico. Bol Asoc Med P R. 2003;95:17–20 . 15449787

[14] Wuthiekanun V, Limmathurotsakul D, Chantratita N, Feil EJ, Day NP, Peacock SJ. *Burkholderia pseudomallei* is genetically diverse in agricultural land in Northeast Thailand. PLoS Negl Trop Dis. 2009;3:e496. 10.1371/journal.pntd.0000496 19652701 PMC2713400

[15] Doker TJ, Sharp TM, Rivera-Garcia B, Perez-Padilla J, Benoit TJ, Ellis EM, Contact investigation of melioidosis cases reveals regional endemicity in Puerto Rico. Clin Infect Dis. 2015;60:243–50. 10.1093/cid/ciu764 25270646

